# Editorial: Advances in Liver Inflammation and Fibrosis Due to Infectious Diseases

**DOI:** 10.3389/fimmu.2020.01760

**Published:** 2020-08-05

**Authors:** Sergio C. Oliveira, M. Victoria Delpino, Guillermo Hernán Giambartolomei, Jorge Quarleri, Gary Splitter

**Affiliations:** ^1^Department of Biochemistry and Immunology, Federal University of Minas Gerais, Belo Horizonte, Brazil; ^2^Facultad de Farmacia y Bioquímica, Instituto de Inmunología, Genética y Metabolismo (INIGEM), Universidad de Buenos Aires, Buenos Aires, Argentina; ^3^Consejo Nacional de Investigaciones Científicas y Técnicas (CONICET), Buenos Aires, Argentina; ^4^Facultad de Medicina, Instituto de Investigaciones Biomédicas en Retrovirus y Sida (INBIRS), Universidad de Buenos Aires (UBA), Buenos Aires, Argentina; ^5^Department of Pathobiological Sciences, University of Wisconsin-Madison, Madison, WI, United States

**Keywords:** fibrosis, HSC, liver, inflammasome, kupffer

Liver inflammation is a common trigger of hepatic disease, and it is considered the main driver of tissue damage (1). Although the liver is able to regenerate, chronic liver inflammation leads to tissue damage with concomitant fibrosis, frequently leading to cirrhosis, and carcinogenesis (2).

The etiology of chronic liver inflammation may be infectious or not. Several microorganisms including bacteria, parasites, fungi, and virus could be involved. Although, other metabolic and immune disorders may also participate. Several proinflammatory cytokines including IL-1α, IL-1β, TNF-α, and IL-6 has been involved in liver disease (3). They are produced by resident macrophages (Kupffer cells) and also by recruited macrophages and neutrophils (4). The common mediators of fibrosis are IL-10 family cytokines, VEGF, EGF, and TGF-β that constitute the main profibrotic cytokines (5). TGF-β stimulate hepatic stellate cell trans-differentiation from a quiescent, vitamin A storing cell to a proliferative myofibroblast that is the central driver of fibrosis (6).

In this Research Topic, a series of articles provide new insights about the current view of liver pathology, inflammation and fibrosis, with original articles about the role of myeloid suppressor cells in fulminant hepatitis, inflammasome activation in brucellosis, new insights in preventing NALDF progression, and the role of NLRP6 in granuloma formation and liver disease associated with schistosomiasis.

This Research Topic also features three review articles regarding the role of glucocorticoids in therapy of liver failure, the interaction of liver and immune cells in brucellosis, the role of T cells in hepatic schistosomiasis, as well as the role of beneficial bacterial in liver disease progression. Also included is a perspective article with proposed mechanisms of interaction between human immunodeficiency virus (HIV) and Kupffer cells.

The original research articles included in this Research Topic were performed involving both *in vitro*, and *in vivo* experiments in the murine model. Such model has acquired particular relevance during the study of non-invasive biomarkers able to assess fibrosis in patients with chronic liver disease.

The liver is the most commonly affected organ in patients with active brucellosis. Accordingly, clinical and biochemical records of liver involvement have been observed in up to 50% of patients with active disease (7). However, the molecular mechanisms involved in liver damage has been recently started to be elucidated. Giambartolomei and Delpino report current understanding of the interaction between liver structural cells including hepatocytes and hepatic stellate cells and immune system cells during *Brucella* infection. They highlight the role of the type IV secretion system (T4SS) and the effector protein BPE005 in the activation of hepatic stellate cells to induce fibrosis. It is likely that BPE005 could participate in granuloma formation that might act as a reservoir of bacteria contributing to the disease chronicity. Prior to the development of liver fibrosis may occur events of inflammation and cell death. The contribution of the inflammasomes activation due to *B. abortus* infection on liver fibrosis was explored in an original article. Arriola Benitez et al. demonstrate in a series of experiments performed using the hepatic stellate cell line LX-2, that inflammasomes NLRP3 and AIM2 are involved in the induction of a fibrotic phenotype in hepatic stellate cells during *B. abortus* infection in a mechanism that involved IL-1β. These experiments were further corroborated using knock out (KO) mice, and strongly suggest the main contribution of this inflammasomes in the liver fibrosis during *B. abortus* infection.

In the liver fibrosis induced by *Schistosoma mansoni* infection, inflammasomes has also involved. Sanches et al. have unraveled using the murine model, the crucial role of the NLRP6 inflammasome in the schistosomiasis-associated hepatic granulomas. Hence, the absence of NLRP6 correlates with a significant reduction in inflammation and collagen deposition, as well as α-SMA and IL-13 levels as fibrotic markers. Both articles stand out the role of inflammasomes as central contributors to liver fibrosis triggered by infectious hepatic diseases. Moreover, parasite worms such as *Schistosoma mansoni* and *S. japonicum* induce a dramatic granulomatous response in liver and intestines. Subsequently, infection may further develop into significant fibrosis and portal hypertension. Zheng et al. extensively review previous reports to elucidate the contribution of T lymphocytes and their secreted cytokines in the immunopathology of schistosomiasis. They clearly indicate the different roles developed by the T-cell subsets for regulating the pathological progression of schistosomiasis in the local microenvironment. Moreover, they highlight recent findings about Tfh and Th9 cells as promoters of liver granulomas and fibrogenesis.

The tumor progression locus 2 (TPL2) is a serine/threonine kinase acting as a key mediator in liver and systemic metabolic disorders with an inflammatory component (8). However, the function of TPL2 in regulating hepatocyte function and liver inflammation during fulminant hepatitis is poorly understood, Xu et al. shed light on the TPL2 role associated with the myeloid derived suppressor cells (MDSC)-mediated protection against fulminant hepatitis. Using the murine model, they demonstrate that TPL2 deficiency suppresses IL-25-induced chemokine CXCL1/2 expression in hepatocytes, thus impairing MDSC recruitment into the liver, and increasing the infiltrate CD4^+^ lymphocyte proliferation that enhances fulminant hepatitis development. These findings strongly suggest that TPL2 is a potential target for the fulminant hepatitis treatment since it plays a critical role in MDSC recruitment.

The interaction between HIV and liver cell population has been previously explored. However, the relevance is still unclear in well-controlled patients on ART. In this context, the perspective article, Zhang and Bansal propose that during HIV infection, changes in the biology of Kupffer cells would create a microenvironment that drives hepatic pathology during microbial translocation. Targeting this pathway could help to improve liver-related consequences in HIV patients.

Liver damage associated to sterile inflammation was evaluated in a model of Non-alcoholic fatty liver disease (NAFLD). This pathology is associated to an excessive storage of fatty acids in the form of triglycerides in hepatocytes and it is considered the main causes of cirrhosis and major risk factors for hepatocellular carcinoma and liver-related death. Wang et al. reveal in a mice model that perforin regulates the abundance of hepatic IFN-γ-producing CD4^+^ T cells with concomitant decrease of macrophage accumulation in liver. This indicate that perforin can act as an immune regulator to prevent NAFLD, suggesting its potential use to prevent hepatic steatosis and other related liver metabolic disorders.

Finally, the last two review article focus on the potentials therapy against liver pathology.

Excessive systemic inflammation is considered as the trigger of liver failure. Glucocorticoids (GCs) can rapidly suppress excessive inflammatory reactions and immune response. In a review article, Xue and Meng describe the current knowledge regarding glucocorticoid therapy in liver failure including emerging information. Although, the topic was addressed from basic research and clinical trials, the current understanding remains inconclusive for the application of GS treatment during liver failure.

The beneficial effect of probiotics has been extended to liver function in cirrhosis, nonalcoholic fatty liver disease, and alcoholic liver disease. Parker et al. review the current knowledge of bacteria of the genus *Alistipes* on its protective role during liver fibrosis among other diseases in animal models.

In summary, this Research Topic highlights the immunopathology of liver damage due to different pathologies. Knowledge acquired from articles contained in this special issue are relevant to the discovery of new targets for controlling liver inflammation and fibrosis.

We wish to express our appreciation to all the authors and reviewers who have participated in this Research Topic.

## Author Contributions

All authors listed have made a substantial, direct and intellectual contribution to the work, and approved it for publication.

## Conflict of Interest

The authors declare that the research was conducted in the absence of any commercial or financial relationships that could be construed as a potential conflict of interest.

## References

[1] Rockey DC Bell PD Hill JA. Fibrosis–a common pathway to organ injury and failure. N Engl J Med. (2015) 372:1138–49. 10.1056/NEJMra1300575 25785971

[2] Tsochatzis EA Bosch J Burroughs AK. Liver cirrhosis. Lancet. (2014) 383:1749–61. 10.1016/S0140-6736(14)60121-5 24480518

[3] Zhangdi HJ Su SB Wang F Liang ZY Yan YD Qin SY. Crosstalk network among multiple inflammatory mediators in liver fibrosis. World J Gastroenterol. (2019) 25:4835–49. 10.3748/wjg.v25.i33.4835 31543677 PMC6737310

[4] Tacke F Zimmermann HW. Macrophage heterogeneity in liver injury and fibrosis. J Hepatol. (2014) 60:1090–6. 10.1016/j.jhep.2013.12.025 24412603

[5] Bataller R Brenner DA. Liver fibrosis. J Clin Invest. (2005) 115:209–18. 10.1172/JCI24282 15690074 PMC546435

[6] Puche JE Saiman Y Friedman SL. Hepatic stellate cells and liver fibrosis. Compr Physiol. (2013) 3:1473–92. 10.1002/cphy.c120035 24265236

[7] Colmenero JD Reguera JM Martos F Sanchez-De-Mora D Delgado M Causse M. Complications associated with Brucella melitensis infection: a study of 530 cases. Medicine (Baltimore). (1996) 75:195–211. 10.1097/00005792-199607000-00003 8699960

[8] Gong J Fang C Zhang P Wang PX Qiu Y Shen LJ. Tumor progression locus 2 in hepatocytes potentiates both liver and systemic metabolic disorders in mice. Hepatology. (2019) 69:524–44. 10.1002/hep.29820 29381809

